# Developing an Ownership Model for Experiential Learning of Social Determinants of Health for Medical Residents

**DOI:** 10.31486/toj.24.0120

**Published:** 2025

**Authors:** Abdullah Noor, Karam Rahat, Koshy George, Luis Capuchina, Brent Ruiz, Matthew Broussard, Yvens Laborde

**Affiliations:** ^1^ Department of Internal Medicine, Ochsner Clinic Foundation, New Orleans, LA; ^2^ The University of Queensland School of Medicine, Ochsner Clinical School, New Orleans, LA; ^3^ Education and Community Outreach Program Management Office, Ochsner Clinic Foundation, New Orleans, LA

**Keywords:** *Community health services*, *health education*, *health services accessibility*, *internship and residency*, *social determinants of health*

## Abstract

**Background:**

Historically, community engagement activities for Ochsner Health internal medicine residents were primarily task-based, involving opportunities to participate in a food drive or a community education project. As an alternative approach, we aimed to create a model that reflected residents’ collective interests, could be meaningfully linked to a community issue, and fostered ownership and sustainability in community engagement.

**Methods:**

This project was conducted at Ochsner Medical Center in the internal medicine residency program during the 2022-2023 academic year with ambulatory clinic groups A, B, and C. In September 2022, we asked each group to respond to the following prompt: “Please identify an issue that you feel most passionate about and would like to contribute to.” Once we identified the issues of interest based on responses and group discussion, we partnered with the community-based organization Giving Hope Foundation New Orleans to plan and carry out projects reflecting resident interests.

**Results:**

Clinic Group A and Group B selected promoting health education as their project and provided one-on-one education based on a resident-prepared health brochure. Five residents participated in the education outreach project for a total of 40 educational encounters. For their project, clinic Group C organized a health fair to promote health education, disease screening, and access. The health fair stations included blood pressure and body mass index screening, nutritional counseling, Medicare/Medicaid application assistance, and cardiopulmonary resuscitation training. The total number of encounters during the event was 100.

**Conclusion:**

Our model showed the possibility of facilitating ownership in the community engagement process among medical residents. However, sustainability depends on replication and incorporation into the residency curriculum.

## INTRODUCTION

Enhancing medical residents’ experiential knowledge of the social determinants of health is an important aspect of addressing concerns about health equity.^1,2^ Consequently, exploring sustainable approaches to integrating such experiential knowledge during training is necessary. Borrowing from the World Health Organization, we understood sustainability to be a project's long-term ability to function with strong community ownership, integration, and resource mobilization.^3^ For medical residents, sustainable community engagement to improve social determinants of health means identifying community needs, finding reliable partners, and developing continuing relationships with clear role determination and accountability to address community needs.^4^ Importantly, the process and implementation need to be owned by the medical residents.

Historically, community engagement in the Ochsner Health internal medicine residency program was primarily task-based. A few individuals would plan specific service events or projects, and other medical residents would volunteer for various tasks. For example, a resident might announce an opportunity to participate in a food drive or a community education project, and the project leader would assign participating residents specific tasks to complete. This task-based model ensured project completion but often overlooked the residents’ interests and passions that could be meaningfully linked to a community issue. Consequently, the model failed to instill a sense of collective ownership in a relationship-building process with community partners.

We aimed to create an ownership model that would enable medical residents to engage with the community in a way that the residents could sustain in partnership with local community organizations. Specifically, our goal was to develop a community engagement process that identifies residents’ collective interests in addressing an issue of social/community concern, facilitates partnership with a community organization to address the concern, and fosters residents’ collective ownership in the community engagement process.

## METHODS

This project was conducted at Ochsner Medical Center in the internal medicine residency program during the 2022-2023 academic year. The internal medicine residency program has a 4+2 model, with 4 weeks spent in the inpatient setting and 2 weeks in the clinic. A 4-hour didactic session is held each week during a clinic block. Because the clinic block rotation allows for free weekends during residency training, the clinic block is the most suitable time for residents to engage with the community.

The residents were divided into 3 clinic groups: A, B, and C. In the 2022-2023 academic year, the program had 56 categorical internal medicine residents. During the clinic blocks in September 2022, we asked each group of residents to respond to the following prompt: “Please identify an issue that you feel most passionate about and would like to contribute to.” The response choices were 5 social determinants of health identified in a 2021 Greater New Orleans community health needs assessment: food insecurity, health literacy, housing insecurity, technological access, and transportation.^5^ Subsequent discussions were held to explore residents’ interests qualitatively and reach a consensus among residents on what issues they most wanted to address. During discussion sessions, the residents could choose issues other than the choices given in the survey. Because our issue determination process involved both the survey and various qualitative discussions that often involved small-group talks, we intentionally decided not to track the number of residents participating in each session.

**Figure 1.**
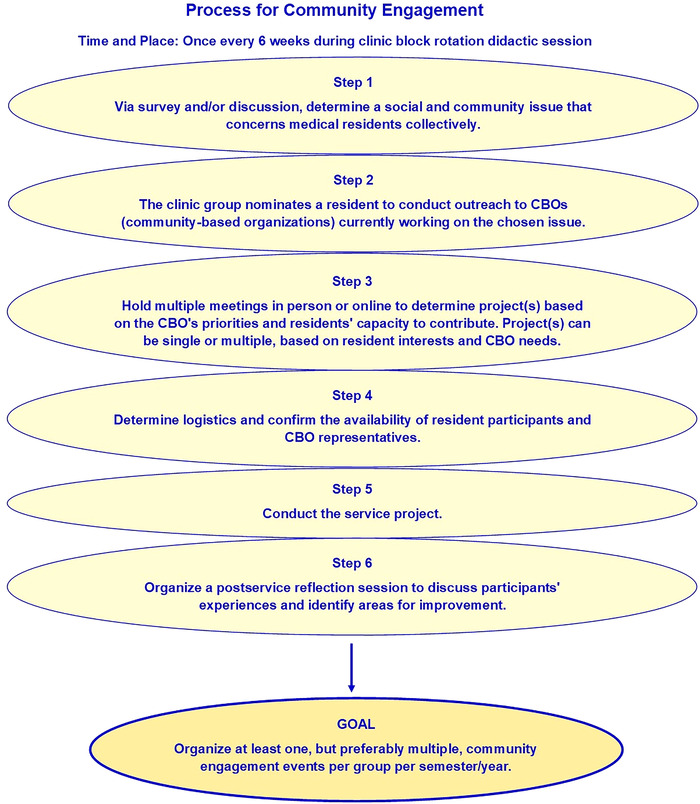
A step-by-step process for community engagement.

Once each resident group identified the issue that was most important to them, the next step was to establish a partnership with a community-based organization currently working on that social determinant. Projects could then be determined and conducted based on the organization's priorities and residents’ contribution capacity. To guide the initiative, we developed a step-by-step process for community engagement (Figure 1).

## RESULTS

### Determining Resident Interests and Establishing Partnerships

Based on survey results and group discussions, the clinic groups chose the following interests:

- Group A: Health education
- Group B: Health education
- Group C: Health education, disease screening, and access

Each clinic group nominated resident leaders. The leaders, in collaboration with mentoring faculty, contacted Second Harvest Food Bank, a nonprofit dedicated to addressing the issue of food insecurity in the Greater New Orleans area. Representatives of Second Harvest Food Bank suggested working with the Giving Hope Foundation New Orleans. The Giving Hope Foundation is a tier 1 partner agency with Second Harvest and operates a food pantry where food is distributed directly to community members.^6^

The Ochsner Medical Center internal medicine residents established a partnership with the Giving Hope Foundation, and after multiple discussions in person and online with leaders of Second Harvest Food Bank and the Giving Hope Foundation, the residents understood that their community partners were interested in promoting health education about hypertension, diabetes, and preventive care. Group C residents proposed to organize a health fair to promote health education and disease screening and to improve access, and the Giving Hope Foundation leadership agreed to collaborate in the health fair.

### Planning and Conducting Projects

Two projects were undertaken in collaboration with the community partners.

#### Group A and Group B Health Education Project.

**Figure 2.**
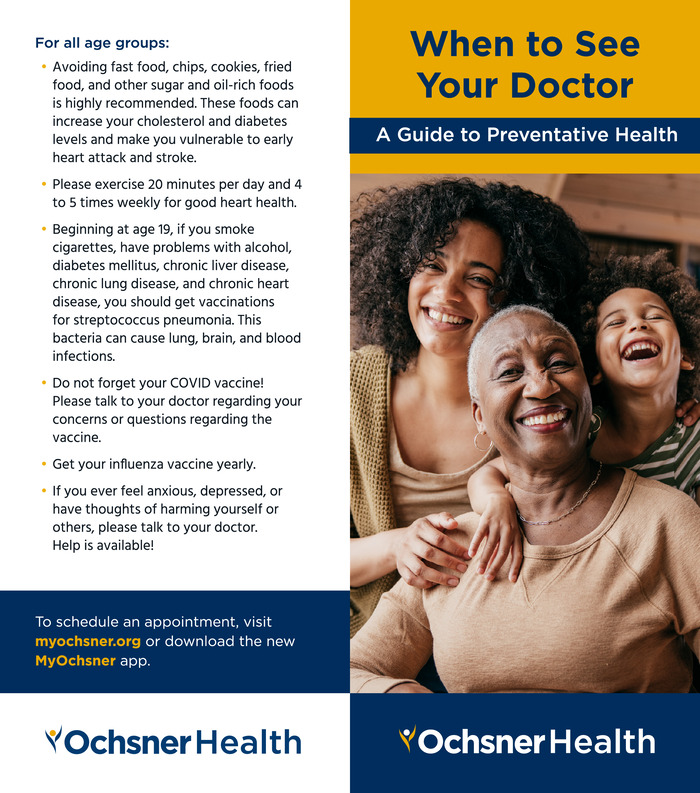
Sample page from the health education handout developed by Ochsner Health internal medicine residents.

A Second Harvest Food Bank leader suggested that a simple yet effective way to provide health education would be to create an information handout and provide education based on the handout. Based on her suggestion, the residents created “When to See Your Doctor: A Guide to Preventative Health” (Figure 2). The handout offered general lifestyle advice suitable for all age groups, including information about influenza and COVID vaccinations, as well as targeted guidelines for individuals aged 18 to 45 years and 45 to 75 years on topics such as managing cardiovascular risk factors (eg, hyperlipidemia and diabetes), smoking cessation, safe sexual practices, and screening recommendations (colonoscopy, mammogram, and cervical cancer). The Ochsner Health marketing department provided oversight, design services, and financing for the handout.

Each week, the Giving Hope Foundation distributed food Monday through Thursday and on Saturday. The internal medicine residents agreed to conduct their education outreach on the Saturdays of clinic blocks when they had the weekends off during ambulatory rotations.

The education outreach activities were conducted on 3 Saturdays at the Giving Hope Foundation food distribution warehouse, with 1 or 2 residents participating each time. The residents walked to the parking lot or stood at the exit gate to meet with clients as they were entering or leaving the warehouse and distributed the handouts, provided related education, and answered questions. These education outreach events gave residents the opportunity to see the Giving Hope Foundation food pantry operations and to interact with community members in a local setting. The residents interacted with 40 community members, conducting a total of 40 educational encounters.

#### Group C Health Fair.

Group C decided to promote health education, disease screening, and access by organizing a health fair. Leaders of the Giving Hope Foundation recommended holding the health fair at the end of a month because turnout at the food pantry was greater then because people generally ran short of money for food at the end of the month. The internal medicine residents partnered with the Xavier University pharmacy department, Medicare/Medicaid representatives, Ochsner Health emergency medicine residents, and a representative of an Ochsner Health community health center to provide screening for blood pressure and body mass index (BMI), Medicare/Medicaid enrollment assistance, and cardiopulmonary resuscitation (CPR) training. Health fair attendees were also provided information about the Ochsner Health community health center where community members could follow up. The Giving Hope Foundation advertised the health fair via flyers, and the event was held in April 2023 on a food distribution day in an open lot in front of the Giving Hope Foundation food distribution warehouse.

Two Ochsner internal medicine residents, 1 emergency medicine resident, 8 pharmacy students, 1 pharmacy resident, 1 pharmacy fellow, 2 Medicare/Medicaid representatives, 1 representative from the Ochsner Health community health center, 1 Ochsner Medical Center nurse practitioner, 1 internal medicine faculty member, 1 pharmacy faculty member, and 1 emergency medicine faculty member participated in the event. The pharmacy trainees and faculty staffed blood pressure and BMI screening stations and provided nutritional counseling. The emergency medicine team, all of whom were certified in basic life support training, set up the CPR training station. The Medicare/Medicaid representatives set up their station for enrollment. The internal medicine residents and nurse practitioner helped with coordination among different stations, directed community members to various stations, and were available to answer questions.

Twenty community members underwent blood pressure and BMI screening. No hypertensive urgency or emergency was detected among the screened members. Twenty community members received nutritional counseling. Fifteen community members received CPR training. Twenty-nine community members completed Medicaid applications. Sixteen community members received Ochsner Health community health center information, and 2 committed to making an appointment. The total number of encounters was 100.

**Figure 3.**
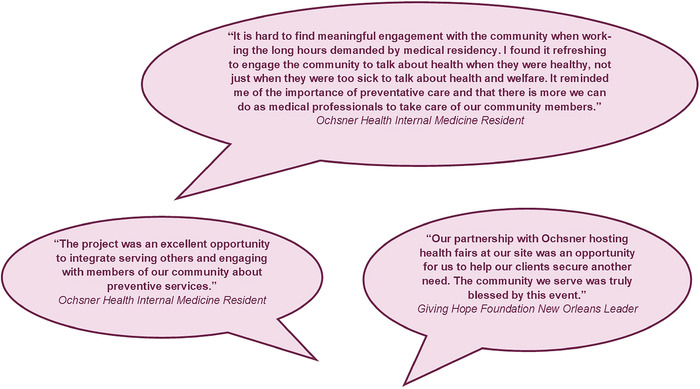
Selected reflections about the health education and health fair projects.

A summary of the projects undertaken by the internal medicine residents is presented in the Table, and selected reflections about the projects are provided in Figure 3.

**Table.** Overview of Ochsner Health Internal Medicine Residency Program Projects and Impact, 2022-2023 Academic Year

Clinic Group	Issues of Interest	Partners	Projects	Impact
A and B	Health education	Giving Hope Foundation New Orleans	Created a preventive health information handout Distributed the handout and provided one-on-one education based on the information in the handout	40 educational encounters
C	Health education, disease screening, and access	Giving Hope Foundation New Orleans Xavier University pharmacy department Louisiana Medicare/Medicaid representatives Ochsner Health emergency medicine residency program Ochsner Health community health center	Organized a health fair involving • BP and BMI screening • Medicare/Medicaid enrollment • CPR training • Nutritional counseling • Community health center information	20 BP and BMI screenings 20 nutritional consultations 29 Medicaid application completions 15 CPR trainings 16 received information about the community health center

BMI, body mass index; BP, blood pressure; CPR, cardiopulmonary resuscitation.

## DISCUSSION

Medical residents face time constraints and have minimal flexibility to participate in meaningful community engagement projects. The lack of time and flexibility substantially limits experiential learning opportunities related to social determinants of health. Our model aimed to gauge the interests and passions of residents about understanding the issues and how they would like to contribute. Then, based on collective interests, we partnered with a community-based organization pursuing a similar goal. In our model, the workload can be divided among group members because the engagement is based on shared mutual interests. Group leaders can coordinate among residents and community partners to achieve agreed-upon goals. For example, for the Group A/Group B project on health education, 1 or 2 residents attended each project activity day, and no resident attended more than 1 day. However, the total number of educational encounters for the 3-day project period was 40. Thus, this approach enables collectively addressing an issue of community concern without burdening a single resident.

While Ochsner Health had established relationships with community partners, the internal medicine residency did not have an adequate structure for residents to pursue meaningful community engagement. No faculty members in residency leadership positions were consistently interested in community engagement. The community engagement curriculum was limited to 1 to 2 lectures annually without any experiential opportunities. Such a lack of faculty support is likely the reality for many internal medicine residency programs, particularly those with limited resources and inadequate faculty interested in community engagement. Our resident-driven model assumes minimal institutional support, particularly in the beginning phase. Once residents have identified their interests, they can connect with community-based organizations and relevant resources. Although faculty members helped facilitate connections, project development and implementation were resident-driven. Residents initiated the meetings with community partners and took ownership to implement the projects. Thus, the model differs from the model of Ahmed et al or the stacked model of Johnston et al that assumes or requires robust institutional support, which may not be a reality for many community programs in resource-deprived settings.^7,8^

However, our approach requires at least a few interested residents and faculty who can gauge the collective interests of the resident group and keep the rest of the residents engaged. Most medical residents are trained to carry out a highly structured cognitive process and often work in hospital or clinic settings. This limited exposure during a crucial period of physician training can hinder residents’ engagement with the historic and socioeconomic realities that contribute to poor health in their communities. Consequently, the definition of “help” may be confined to perfectly following an algorithm or quickly sending patients home. Our model attempts to provide a process for residents to engage with each other and with community members in community settings, but success relies on having a few residents taking ownership to facilitate the process.

### Limitations and Recommendations

One limitation of our project is that only 5 residents from Group A/Group B participated in the education outreach activities and only 2 residents from Group C participated in the health fair. One explanation is that the health fair was held near the end of the academic year (April 2023) when many residents, particularly third-year residents, are transitioning to the next phase of their careers. Furthermore, because the internal medicine residency program had no sustained community engagement activities before this project was initiated, appropriate discourse—both formal and informal discussion among residents—regarding community engagement and its relevance to their careers had not been developed. Multiple replications of our process for community engagement may help increase participation in future years.

Furthermore, sustainability could be ensured by incorporating the program as an integral part of the ambulatory curriculum. Because the program is currently not a mandatory part of the curriculum, a great deal of commitment and personal initiative is required on the part of the residents. We had to adapt the program in the 2023-2024 academic year by aligning with the Ochsner Health community engagement team that provided internal medicine residents with a calendar of events that allowed them to volunteer and have experiential community learning opportunities based on their interests and availability. However, the community engagement team modification lacked the coordinated effort of the prior year project. We recommend that future initiatives focus on integrating community engagement into the internal medicine curriculum to ensure sustainability.

## CONCLUSION

We developed a community engagement model for medical residents that builds on collective interests and collaborative effort. The model aimed to promote resident ownership and assumed minimal institutional support. Such an approach may apply to residency programs operating in resource-deprived settings that may not have dedicated faculty involved in community engagement. Our projects illustrated the application of the model in real-life conditions. Replicating such a model and incorporating the process in the internal medicine residency curriculum will help ensure longevity and success.

## Notes

*This article meets the Accreditation Council for Graduate Medical Education and the American Board of Medical Specialties Maintenance of Certification competencies for Patient Care, Medical Knowledge, Interpersonal and Communication Skills, and Professionalism.*

## References

[1] 2023 National Healthcare Quality and Disparities Report. Agency for Healthcare Research and Quality. December 2023. Last reviewed May 2024. Accessed March 21, 2025. ahrq.gov/research/findings/nhqrdr/nhqdr23/index.html 38377267

[2] Thornton RL, Glover CM, Cené CW, Glik DC, Henderson JA, Williams DR. Evaluating strategies for reducing health disparities by addressing the social determinants of health. Health Aff (Millwood). 2016;35(8):1416-1423. doi: 10.1377/hlthaff.2015.1357 27503966 PMC5524193

[3] African Programme for Onchocerciasis Control Management. Guidelines for conducting an evaluation of the sustainability of CDTI projects. World Health Organization. September 2004. Accessed March 21, 2025. iris.who.int/bitstream/handle/10665/337078/337078-eng.pdf

[4] Improving Social Determinants of Health—Getting Further Faster. U.S. Centers for Disease Control and Prevention. March 2023. Accessed December 12, 2025. cdc.gov/health-equity-chronic-disease/media/pdfs/2024/04/GFF-eval-brief-508.pdf

[5] Louisiana Public Health Institute. Greater New Orleans Area: 2021 Community Health Needs Assessment. LCMC Health–Ochsner Health. December 2021. Accessed March 7, 2025. lcmchealth.org/sub/59170/documents/2021_Community_Health_Needs_Assessment_UMC.pdf

[6] Giving Hope Foundation New Orleans. Accessed April 11, 2025. givinghopenola.org

[7] Ahmed SM, Neu Young S, DeFino MC, Franco Z, Nelson DA. Towards a practical model for community engagement: advancing the art and science in academic health centers. J Clin Transl Sci. 2017;1(5):310-315. doi: 10.1017/cts.2017.304 29707251 PMC5915810

[8] Johnston B, Ruffalo L, Nelson D, O’Connor S, Petterson E, Young S. The stacked community engagement model: a practical model for developing community-engaged academic medical faculty. J Clin Transl Sci. 2023;7(1):e36. doi: 10.1017/cts.2023.1 36845313 PMC9947615

